# Gpr83 expression is not required for the maintenance of intestinal immune homeostasis and regulation of T-cell-dependent colitis

**DOI:** 10.1111/j.1365-2567.2008.02857.x

**Published:** 2008-11

**Authors:** Christy Toms, Heidi Jessup, Claire Thompson, Dilair Baban, Kay Davies, Fiona Powrie

**Affiliations:** 1 Sir William Dunn School of Pathology, University of Oxford Oxford, UK; 2 Amgen Inc. Seattle, WA, USA; 3 Genomics Group, The Wellcome Trust Center for Human Genetics Oxford, UK; 4 MRC Functional Genetics Unit, Department of Physiology, Anatomy and Genetics, University of Oxford Oxford, UK

**Keywords:** inflammatory bowel disease, mucosal immunity, regulatory T cells, transgenics/knockouts

## Abstract

Regulatory T (T_R_) cells are integral to the maintenance of intestinal homeostasis, where an intricate balance between tolerance and immunity must be maintained. Recently, studies have focused on the identification of molecules involved in the function and/or development of T_R_ cells. One such molecule, the G-protein coupled receptor Gpr83, has been identified through gene expression analysis as being overexpressed within thymic and peripheral naturally arising regulatory T (nT_R_) cell populations. The aim of this study was to further define the characteristics of Gpr83 expression and to investigate the role of Gpr83 in T_R_-cell development and function through the generation and analysis of Gpr83-deficient mice. Following activation, naïve CD4^+^ T cells induce Gpr83 expression in a transforming growth factor (TGF)-β dependent manner. Rather than being a general marker of activation, Gpr83 expression could only be detected in cells also expressing forkhead/winged helix transcription factor (Foxp3), further supporting the association of Gpr83 with the regulatory cell phenotype. Mice deficient in Gpr83 expression developed normally and did not display signs of inflammatory disease. Thymic nT_R_-cell development was unaffected by a lack of Gpr83 expression and peripheral nT_R_-cell homeostasis was normal when compared with that of wild-type mice. Gpr83 expression was dispensable for the regulatory activity of nT_R_ cells as Gpr83-deficient nT_R_ cells could suppress the development of disease in a T-cell transfer model of colitis. These results suggest a redundant role for Gpr83 in the function of T_R_ cells in this model of disease. Further studies are required to determine the role of Gpr83 in T_R_-cell biology.

## Introduction

Regulatory T (T_R_) cells are essential for the maintenance of immunological homeostasis and the effective regulation of immune responses within the body. Given the therapeutic potential of these cells, extensive studies over the last decade have been carried out with the aim of understanding the developmental and functional aspects of T_R_-cell biology. The most widely studied of these populations are the naturally arising regulatory T (nT_R_) cells which develop within the thymus and are characterized by their expression of the forkhead/winged helix transcription factor (Foxp3) and CD25.^1^–^4^ More recently, accumulating evidence suggests the potential for the extrathymic development of regulatory T-cell populations.^5^–^8^ The mechanisms by which T_R_ cells mediate their suppressive activity have still to be clearly defined and it is likely that multiple pathways are utilized depending on tissue specificity and the type of immune response being regulated.

In the intestine, the balance between immunity and tolerance is crucial for maintaining the ability to distinguish and respond appropriately to the vast array of dietary antigens, commensal bacteria and harmful pathogens encountered within the gut-associated lymphoid tissue. The importance of T_R_ cells in maintaining this balance is highlighted by studies of human patients with immunodysregulation polyendocrinopathy enteropathy X-linked (IPEX) syndrome.^9^ In these patients, mutations in the transcription factor Foxp3 result in a lack of functional nT_R_ cells and a primary target of the resulting multi-organ inflammation is the gut. Animal models of colitis have been used to investigate the mechanisms involved in mediating the prevention of intestinal inflammation. Molecules such as transforming growth factor (TGF)-β, interleukin (IL)-10 and cytotoxic T-lymphocyte antigen (CTLA)-4 have all been implicated as playing a role in the suppression of colitogenic T-cell activity mediated by nT_R_ cells,^10^–^13^ although the exact cell types producing and responding to these immune modulators are still unclear. More recently, specialized subsets of mucosal dendritic cells have been shown to induce the conversion of antigen-specific T cells into Foxp3^+^ regulatory cells in a retinoic acid and TGF-β dependent manner,^14^,^15^ providing evidence to suggest that induced as well as natural T_R_ cells act in concert to regulate intestinal immunity.

Gene expression profiling of CD4^+^ Foxp3^+^ T_R_ cells has provided valuable insights into the development and function of these cells.^16^–^19^ One molecule to emerge from these studies is the G-protein coupled receptor Gpr83, which displays highly restricted expression within the nT_R_-cell subset. Of particular interest is the potential use of Gpr83 as a marker of nT_R_ cells as a consequence of its proposed cell surface expression. Gpr83 was originally identified as a glucocorticoid-responsive gene in a murine thymoma cell line^20^ and subsequently shown to display high expression levels within the brain.^21^

Whilst the association of Gpr83 with both thymic and peripheral T_R_-cell subsets has been established, the functional role of Gpr83 remains unclear. Gene expression studies reveal a clear link between Gpr83 expression and Foxp3.^22^ Furthermore, retroviral introduction of Foxp3 into naïve CD4^+^ T cells is sufficient to induce high levels of Gpr83 expression as measured at the mRNA level.^19^,^23^ More recently, Hansen *et al.* demonstrated an enhanced ability to induce Foxp3 in cells retrovirally transduced with Gpr83.^23^ The physiological significance of this, however, remains to be determined. In this study we have generated mice deficient in Gpr83 expression to further characterize the role of this protein in T_R_-cell biology.

## Materials and methods

### 

#### Mice

BALB/c, BALB/c Gpr83^−/−^, C57BL/6/129 Foxp3^GFP^, C57BL/6 dnTGF-βRII, C57Bl/6, BALB/C Ly9·2 and BALB/c Ly9·2 RAG2^−/−^ mice were maintained in specific pathogen-free conditions at the Sir William Dunn School of Pathology (University of Oxford, Oxford, UK). Experiments were conducted in accordance with the UK Scientific Procedures Act 1986.

#### Purification of CD4^+^ T-cell subsets

CD4^+^ T cells were purified from spleens by negative depletion using rat anti-mouse CD8 (clone YTS 169), B220 (RA3-6B2), major histocompatibility complex (MHC) class II (TIB 120) and the macrophage marker Mac-1 (M1-70) (all purified from hybridoma supernatants by affinity chromatography), together with anti-rat coated Dynabeads (Invitrogen, Paisley, UK). CD4 cells were enriched from the thymus by negative depletion using anti-CD8 antibodies and anti-rat coated Dynabeads. Purified CD4^+^ splenocytes were stained with anti-mouse CD4-CyChrome (clone RM4·5; BD Biosciences, Erembodegem, Belgium), anti-mouse CD45RB-fluorescein isothiocyanate (FITC) (clone 16A; BD Biosciences) and anti-mouse CD25-phycoerythrin (PE) (clone PC61; BD Biosciences). For purification of CD4 single positive (SP) CD25^+^ and CD25^−^ populations, CD4-enriched thymocytes were stained with CD4-Cyc, CD25-PE and CD8-allophycocyanin (APC) (clone 53-6·7; BD Biosciences). Subpopulations of CD4^+^ cells were sorted using a MoFlo (DakoCytomation, Ely, UK) cell sorter. Purified populations were at least > 98·5% pure upon re-analysis.

#### T-cell stimulation cultures

Purified splenic CD4^+^ CD45RB^hi^ CD25^−^ [or green fluorescent protein (GFP)^−^] naïve T cells (1 × 10^6^/ml) were activated with either plate-bound anti-CD3 (145-2c11) and anti-CD28 (35·51) (both at 10 μg/ml) or in some instances with anti-CD3 and anti-CD28 coated beads (Invitrogen). Cells were cultured in complete RPMI (Invitrogen) containing 10% fetal calf serum (FCS), 2 mm l-glutamine, 0·05 mm 2-mercaptoethanol and 100 U/ml each of penicillin and streptomycin. Where indicated, cells were cultured in the presence of 2 ng/ml recombinant human TGF-β (R&D Systems, Abingdon, UK).

#### RNA isolation and quantitative polymerase chain reaction (PCR)

RNA was isolated from cells using the RNA Plus Mini Kit (Qiagen, Crawley, UK) and cDNA synthesized using Superscript III (Invitrogen). Quantitative PCR was performed using CD3 gamma chain (CD3γ), Gpr83 and Foxp3 specific primers (QuantiTect Primer Assays, Qiagen) and Platinum SYBR Green qPCR Supermix (Invitrogen). cDNA samples were assayed in triplicate using the Chromo4 detection system (GMI, Ramsey, MN), and gene expression levels for each individual sample were normalized to CD3γ. Mean relative gene expression was determined and expressed as 2^−ΔCT^. In some instances, Taqman quantitative PCR analysis was performed. First-strand cDNA was synthesized using TaqMan Reverse Transcription reagents (Applied Biosystems, Foster City, CA) according to the manufacturer’s instructions using random hexamers. Samples were distributed on plates at 20 ng/well and run in triplicate. PCR reactions were set up in 25-μl volumes using TaqMan Universal PCR Master Mix (Applied Biosystems) on an Applied Biosystems 7900HT Sequence Detection System. Cycle threshold (C_T_) values were determined using sequence detector software version 2.2.2 (Applied Biosystems) and transformed to 2^−ΔCT^ for relative expression comparison of genes to hypoxanthine-guanine phosphoribosyl transferase (HPRT).

#### Generation of Gpr83^−/−^ transgenic mice

Mice genetically deficient in Gpr83 were generated by homologous recombination in 129-derived embryonic stem (ES) cells using established protocols (Lexicon Genetics, The Woodlands, TX). In brief, a 2374-bp genomic region containing exons 2–3 of Gpr83 (NM_010287) and encoding amino acids 128–215 of the mature polypeptide was deleted by germline cre-mediated excision of a floxed allele. Correct gene targeting and excision were confirmed using Southern blot and PCR analyses. Mice homozygous for the Gpr83 mutation were generated at the expected frequency from heterozygous intercrosses and did not display any overt phenotype. Genotyping was performed using the following primers: GPR83-1, 5′-CAGAGCCCGAGTTCTTCCCA-3′; GPR83-6, 5′-GGCAGAACC TGGTAACTACTG-3′; and GPR83-11, 5′-GCGATAGCTGTCAGAGTC AGT G-3′. The mutation was moved from a random hybrid C57BL/5:129 genetic background by five successive backcrosses to BALB/c, using Marker-Assisted backcrossing (Charles River, Wilmington, MA). The resulting mice were homozygous for the BALB/c allele of all sequence length polymorphisms in the MaxBax panel.

#### Generation of bone marrow chimeras

Bone marrow was isolated from Ly9.2-BALB/c congenic and Gpr83^−/−^ age-matched mice and mixed at a 1 : 1 ratio. Gamma-irradiated (5.5 Gy, 550 rad) Ly9·2 RAG2^−/−^ recipients were injected with 5 × 10^6^ mixed bone marrow cells/mouse intravenously. 10 weeks after transfer, T-cell reconstitution was assessed by analysis of expression of the *Ly9* allele in peripheral blood.

#### Colitis induction and histological assessment

Naïve CD4^+^ CD45RB^hi^ T cells were isolated from BALB/c wild-type and Gpr83^−/−^ mice and injected intraperitoneally (i.p.) into BALB/c RAG2^−/−^ immunodeficient recipients (4 × 10^5^/mouse). BALB/c wild-type and Gpr83^−/−^ CD4^+^ CD45RB^lo^ CD25^+^ T cells (2 × 10^5^) were co-injected i.p. where indicated. Mice were monitored weekly for wasting disease and any mice losing more than 20% of its starting body weight or showing severe signs of disease were killed. Colons were removed from mice 8–10 weeks after T-cell reconstitution and fixed in 10% formalin. Six-micrometre paraffin-embedded sections were cut and stained with haematoxylin and eosin (H&E) and inflammation was assessed using a modified version of a previously described scoring system.^13^ Colitis was graded semiquantitatively based on four main criteria: (1) the degree of hyperplasia/goblet cell depletion; (2) the extent of lymphocytic infiltration and inflammation; (3) the percentage area affected, and (4) the presence of severe inflammation as assessed by the presence of submucosal inflammation and crypt abscesses. For each criterion, a score of 0–3 was given and the scores for each criterion added to give a final score of 0–12. Three sections for each colon were taken (proximal, mid and distal) and the colonic score represents the average from individual scores for each of the three sections.

## Results

### Gpr83 is induced upon T-cell activation

**Figure 1:**
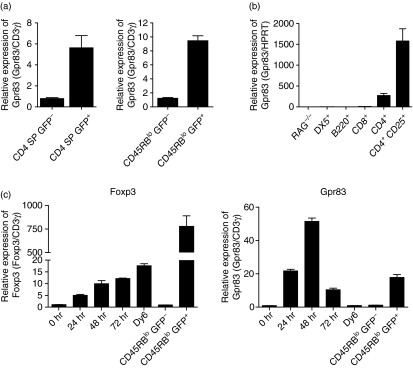
Expression of the G-protein coupled receptor Gpr83 is restricted to the CD4^+^ T-cell compartment and is induced following T-cell activation. (a) T cells were isolated from the thymus and spleen of Foxp3^GFP^ mice and sorted using fluorescence-activated cell sorting (FACS) based on expression of CD4 and green fluorescent protein (GFP) (thymocytes) and CD4, CD45RB and GFP (splenocytes). RNA was isolated from the indicated populations and quantitative PCR (qPCR) used to determine the relative expression of Gpr83. Results shown are from one representative experiment from at least three independent experiments. CD3-gamma (CD3γ) chain mRNA expression was used as an internal standard and relative mRNA amounts were normalized to the GFP^−^ population. Results are expressed as the mean of triplicate wells ± standard error of the mean (SEM). (b) Splenocytes were isolated from BALB/c mice and sorted using FACS based on expression of the indicated markers. Total splenocytes from RAG^−/−^ spleens were also isolated. RNA was isolated from the individual cell subsets and qPCR used to assess expression of Gpr83. Hypoxanthine-guanine phosphoribosyl transferase (HPRT) mRNA expression was used as an internal standard and relative mRNA amounts were normalized to RAG^−/−^ expression. Results are expressed as the mean of triplicate wells ± SEM. (c) CD4^+^ CD45RB^hi^ GFP^−^ T cells were sorted using FACS from spleens isolated from Foxp3^GFP^ mice (purity > 99%). Cells were activated with αCD3 and αCD28 coated beads. At the indicated time-points, cells were harvested and relative expression analysed by qPCR. Freshly isolated CD4^+^ CD45RB^lo^ GFP^−^ and CD4^+^CD45RB^lo^ GFP^+^ T cells were used as indicators of relative Foxp3 and Gpr83 expression levels. CD3γ expression was used as an internal standard and relative amounts were normalized to unactivated (0 hr) T-cell expression. Results shown are the mean of triplicate wells ± SEM. Results are from one representative experiment from at least three independent experiments.

Gpr83 expression profiling has identified Gpr83 as being differentially expressed amongst the CD4^+^ Foxp3^+^ T-cell population relative to the Foxp3^−^ subset. Quantitative PCR analysis confirms this restricted expression in both thymic and peripheral CD4^+^ Foxp3^+^ populations using T-cell subsets isolated from Foxp3^GFP^ transgenic mice^22^ (Fig. 1a). These mice express a GFP-Foxp3 fusion protein which can be used to track expression of Foxp3 and thus the isolation of Foxp3^−^ (GFP^−^) and Foxp3^+^ (GFP^+^) populations by fluorescence-activated cell sorting (FACS). To determine whether Gpr83 is more widely expressed amongst other immune cell populations, leucocytes were isolated from the spleens of wild-type and BALB/c RAG^−/−^ mice and sorted by FACS into various subsets based on the following markers: CD4, CD8, B220 and DX5. Quantitative PCR analysis revealed that Gpr83 expression was only detectable amongst CD4^+^ T cells and, as has been shown previously, this was attributable to the enhanced expression of Gpr83 within the CD4^+^ CD25^+^ population (Fig. 1b). A number of markers identified as being enriched within nT_R_ cells have subsequently been shown to be more closely associated with an activatory rather than regulatory phenotype. To investigate whether Gpr83 expression could be detected following the activation of naïve CD4^+^ T cells we used the Foxp3^GFP^ transgenic mice. This allowed us to isolate a predominantly naïve population that was negative for expression of Foxp3. Splenic CD4^+^ CD45RB^hi^ GFP^−^ naïve T cells were isolated by FACS (purity > 99%) and activated *in vitro* with αCD3/αCD28 coated beads. Expression of Gpr83 could be detected 24 hr after the activation of these cells, as assessed by quantitative PCR, and had returned to baseline by 72 hr (Fig. 1c). At its peak the level of expression detected within the naïve T cells was as high as that seen in *ex vivo* CD45RB^lo^ GFP^+^ nT_R_ cells. Gpr83 appears to be a more acute activation marker as only low levels of GPR83 mRNA were found in the Foxp3^−^ antigen experienced pool (CD4^+^ CD45RB^lo^Foxp3^−^) analysed directly *ex vivo*. In view of the close association observed in T_R_ cells between Foxp3 expression and Gpr83, we also analysed expression of Foxp3 in these cells. Foxp3 mRNA also showed activation-induced induction and was detectable within 24 hr following activation (Fig. 1c). The level of expression in the activated cells was substantially lower than that seen in CD4^+^ CD45RB^lo^ CD25^+^ nT_R_ cells but appeared to be maintained throughout the period of culture. Expression was higher than that observed in the *ex vivo* CD4^+^ CD45RB^lo^ CD25^−^ T cells, suggesting that this transient induction of Foxp3 was not a general facet of antigen-activated T-cell populations. Taken together, these data show that both Foxp3 and Gpr83 can be induced following the activation of CD4^+^ Foxp3^−^ naïve T cells.

### Peripheral induction of Gpr83 is dependent on TGF-β signalling

**Figure 2:**
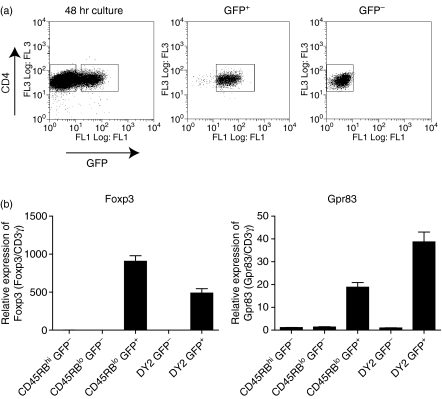
Activation-induced G-protein coupled receptor Gpr83 is restricted to forkhead/winged helix transcription factor (Foxp3)-expressing cells. (a) Naïve CD4^+^ CD45RB^hi^ green fluorescent protein (GFP)^−^ T cells were isolated from Foxp3^GFP^ mice by fluorescence-activated cell sorting (FACS; purity >99%). Cells were activated with plate-bound αCD3 and αCD28 (both 10 μg/ml) for 48 hr in the presence of recombinant human transforming growth factor (TGF)-β (2 ng/ml). Cells were harvested and re-sorted based on expression of CD4 and GFP (CD4^+^ GFP^−^, > 99% purity; CD4^+^ GFP^+^, > 98% purity). (b) RNA was isolated from the sorted populations and quantitative polymerase chain reaction (qPCR) used to determine the relative expression of Foxp3 and Gpr83. CD3γ was used as an internal standard and relative mRNA levels normalized to the CD4^+^ CD45RB^hi^ GFP^−^ starting population. Results shown are representative of three independent experiments and are expressed as the mean of triplicate wells ± standard error of the mean (SEM). WT, wild type.

TGF-β is known to drive the peripheral induction of Foxp3 in activated CD4^+^ T cells and there is evidence that low levels of Foxp3 detected following activation *in vitro* reflect a response to TGF-β in the culture medium. To test whether Gpr83 induction was also dependent on TGF-β, naïve CD4^+^ T cells from mice expressing a dominant negative form of the TGF-β receptor II subunit (dnTGF-βRII), and as such displaying impaired responsiveness to TGF-β, were activated as before and Gpr83 and Foxp3 expression determined by quantitative PCR. As has previously been reported, Foxp3 expression in naïve CD4^+^ T cells was highly dependent on TGF-β signalling as no detectable Foxp3 mRNA was seen in activated dnTGF-βRII CD4^+^ T cells. Analysis of Gpr83 expression in these cells clearly showed that, like Foxp3, induction of Gpr83 expression was dependent on TGF-β signalling as no expression could be detected in dnTGF-βRII activated T cells. CD4^+^ T cells isolated from wild-type mice were able to induce expression of both Foxp3 and Gpr83 and no differences were observed in cell accumulation and viability between wild-type and dnTGF-βRII cultures (data not shown), indicating that the inability to detect expression of Gpr83 and Foxp3 was not a result of a failure to activate these cells, or increased cell death within the culture. Furthermore, as has previously been reported, Foxp3 expression was not reduced in nT_R_ cells isolated from the periphery of dnTGF-βRII mice (Fig. 2b).^11^ However, there was a significant reduction in expression of Gpr83 in dnTGF-βRII nT_R_ cells as compared with wild-type cells. Similar results were seen in CD4^+^ CD25^+^ nT_R_ cells isolated from the thymus of dnTGF-βRII mice when compared with wild-type thymic nT_R_ cells (data not shown). These results reveal that, as with Foxp3, expression of Gpr83 is closely associated with TGF-β.

### Induction of Gpr83 is restricted to Foxp3-expressing cells

**Figure 3:**
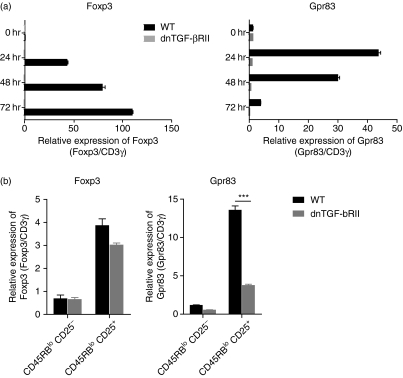
Induction of expression of the G-protein coupled receptor Gpr83 is dependent on transforming growth factor (TGF)-β responsiveness. (a) Naïve CD4^+^ CD45RB^hi^ CD25^−^ T cells were isolated from dnTGF-βRII or aged-matched wild-type mice by fluorescence-activated cell sorting (FACS; purity > 98%) and activated *in vitro* with plate-bound αCD3 and αCD28 (both 10 μg/ml). RNA was isolated at the indicated time-points and forkhead/winged helix transcription factor (Foxp3) and Gpr83 expression analysed by quantitative polymerase chain reaction (qPCR). CD3γ was used as an internal standard and relative mRNA amounts were normalized to unactivated (0 hr) expression levels. Results shown are from one of at least three independent experiments and are shown as the mean of triplicate wells ± standard error of the mean (SEM). (b) Splenic CD4^+^ CD45RB^lo^ CD25^−^ and CD25^+^ populations were isolated from age-matched wild-type and dnTGF-βRII mice. RNA was isolated and qPCR used to determine the relative expression of Foxp3 and Gpr83. As previously, the CD3γ chain was used as an internal standard and relative mRNA levels normalized to the CD4^+^ CD45RB^lo^ green fluorescent protein (GFP)^−^ populations. Results shown are of one of three independent experiments and are expressed as the mean of triplicate wells ± SEM. Student’s *t*-test was used to determine the statistical significance of differences observed (*P*< 0·001).

To further dissect the link between Gpr83 expression and Foxp3, we next asked whether Gpr83 expression was associated with Foxp3-expressing cells or was more a general feature of activated cells. To do this, we again used the Foxp3^GFP^ mice to allow us to start with a Foxp3^−^ naïve CD4^+^ T-cell population. The expression of GFP in Foxp3^+^ cells also allowed us to use FACS to isolate pure populations of Foxp3^−^ and Foxp3^+^ cells for subsequent quantitative PCR analysis of Gpr83. CD4^+^ CD45RB^hi^ GFP^−^ naïve T cells were isolated and activated in the presence of TGF-β (2 ng/ml). After 48 hr, the cells were sorted based on GFP expression (Fig. 3a) and Gpr83 expression determined in the GFP^−^ (Foxp3^−^) and GFP^+^ (Foxp3^+^) fractions. As expected, Foxp3 expression was only detectable in the GFP^+^ fraction, confirming the purity of the cells isolated following sorting. Gpr83 expression was also found to be enriched within the GFP^+^ Foxp3^+^ fraction with little or no expression detectable within the GFP^−^ populations. Similar results were also obtained from naïve CD4^+^ T cells activated in the absence of exogenous TGF-β (data not shown). These results clearly demonstrate that Gpr83 expression is highly enriched within Foxp3-expressing cells, indicating that Gpr83 is not a marker of general T-cell activation, but is tightly linked to Foxp3 expression through a TGF-β-dependent mechanism.

### The generation and characterization of Gpr83-deficient mice

**Figure 4:**
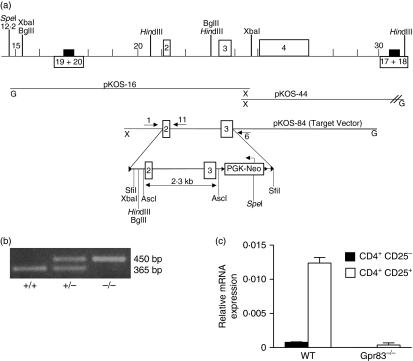
Generation of mice deficient in the G-protein coupled receptor Gpr83. (a) Schematic representation of the targeting construct used to generate mice lacking the Gpr83 allele. Two *loxP* sites were introduced, and the targeting vector designed to delete exons 2 and 3. Chimeric Gpr83 floxed female mice were mated to male protamine Cre mice in order to induce germline Gpr83 deletion. (b) Polymerase chain reaction (PCR) screening of Gpr83-targeted offspring. Primers 1 and 11 amplify a 365-bp region of the endogenous Gpr83 gene. Primers 1 and 6 identify the targeted gene and produce a 450-bp product. (c) Expression of Gpr83 exons 2–3 was assessed by TaqMan in purified CD4^+^ CD25^−^ and CD4^+^ CD25^−^ T-cell populations from Gpr83^−/−^ mice and wild-type (WT) littermate controls (*n*= 3). Hypoxanthine-guanine phosphoribosyl transferase (HPRT) was used as an internal standard. Results represent the mean relative expression from three individual mice ± standard deviation.

To gain an insight into the functional role of Gpr83 in nT_R_-cell development and function we generated Gpr83-deficient mice. A targeting vector was designed to replace exons 2 and 3 of Gpr83 with a neomycin resistance cassette. In Gpr83^−/−^ mice, exons 1 and 4 are joined, followed by an immediate stop codon (Fig. 4a). The predicted protein sequence is truncated after the second transmembrane domain, resulting in a non-functional receptor. Chimeric ‘floxed’ mice were generated and bred to protamine Cre mice to produce mutant mice. PCR screening was used to determine the transmission of the Gpr83-targeted allele (Fig. 4b). To confirm the absence of Gpr83 expression within peripheral nT_R_ cells, CD4^+^ CD25^−^ and CD4^+^ CD25^+^ T cells were isolated from wild-type and Gpr83^−/−^ mice and analysed by quantitative PCR for expression of Gpr83. No expression could be detected in either cell population isolated from Gpr83^−/−^ mice as compared with wild type (Fig. 4c).

### Gpr83 expression is not required for the development or peripheral maintenance of nT_R_ cells

**Figure 5:**
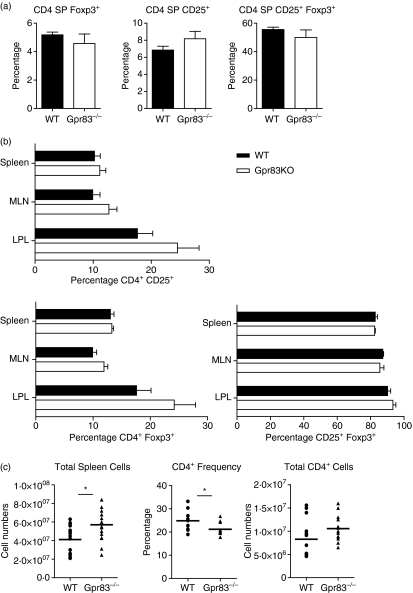
Regulatory T-cell analysis in mice deficient in the G-protein coupled receptor Gpr83. (a) Thymocytes were isolated from Gpr83^−/−^ and age-matched wild-type (WT) BALB/c mice. The percentage of forkhead/winged helix transcription factor (Foxp3) and CD25 expression in CD4 single positive (SP) cells was determined by fluorescence-activated cell sorting (FACS). Results show the mean frequency of the indicated cell population in the total CD4 SP thymocytes (*n*= 3) ± standard deviation (SD). (b) Lymphocytes from WT and Gpr83^−/−^ mice were isolated from the spleen (*n*= 12), mesenteric lymph nodes (MLN) (*n*= 12) and colonic lamina propria (LPL) (*n*= 4). The percentage of Foxp3 and CD25 expression in CD4^+^ T cells was determined by FACS. Results show the mean frequency of the indicated cell population in CD4^+^ T cells ± SD. (c) Individual spleens from WT and Gpr83-deficient mice were analysed for total cellularity, total CD4^+^ cell numbers and CD4^+^ frequency. Mann–Whitney statistical analysis was performed to determine the statistical significance of differences observed. KO, knockout.

To determine whether Gpr83 expression was required for the development of nT_R_ cells, the distribution of CD25 and Foxp3 amongst the CD4 SP thymocytes was assessed by FACS analysis. No differences in total thymic cellularity were detected between wild-type BALB/c age-matched thymi and Gpr83^−/−^ thymi (data not shown). The development of double-negative, double-positive and CD4 SP and CD8 SP populations was also comparable (data not shown). A lack of Gpr83 expression did not affect the development of nT_R_ cells as CD4 SP thymocytes from wild-type and Gpr83^−/−^ mice were equivalent in their expression of CD25 and Foxp3 (Fig. 5a). Furthermore, the frequency of CD4 SP CD25^+^ Foxp3^+^ cells in the CD4 SP CD25^+^ populations was also indistinguishable between wild-type (55·5 ± 1·7%) and Gpr83^−/−^ (49·9 ± 5·3%) cells (Fig. 5a).

We next asked whether there were changes in the peripheral nT_R_-cell population. Lymphocytes were isolated from the spleen, mesenteric lymph nodes (MLNs) and colonic lamina propria and analysed by FACS for expression of CD4, CD25 and Foxp3 (Fig. 5b). Within the spleen, wild-type and Gpr83^−/−^ lymphocytes expressed equivalent frequencies of CD25 and Foxp3 in the CD4 population. The frequency of CD4^+^ CD25^+^ Foxp3^+^ cells was also similar between wild-type (80·6 ± 9·5%) and Gpr83^−/−^ (81 ± 7·45%) cells, indicating no overt activation of T cells was occurring in the absence of Gpr83 expression. Whilst the frequency of T_R_ cells within the spleen appeared normal, there was a modest but significant increase in total spleen cell numbers in Gpr83^−/−^ mice compared with wild type (Fig. 5c). This was associated with a small, but statistically significant reduction in the overall frequency of CD4^+^ T cells, whilst total numbers remained the same. The exact nature of the cell type responsible for this increase in spleen cellularity is unclear as there was no detectable difference in the frequencies of B cells, CD8 cells, dendritic cells, monocytes, macrophages or natural killer (NK) cells (data not shown).

Mice lacking Gpr83 remained healthy. Furthermore, there was no histological evidence of colitis (data not shown), indicating that Gpr83 is not crucial for the maintenance of intestinal homeostasis or the effective functioning of nT_R_ cells within the colon or associated lymph nodes. However, it was still possible that differences in the frequency or distribution of nT_R_ cells could result from a defect in Gpr83 function. Within the MLNs, the frequency of CD4^+^ T cells that were either CD25^+^ or Foxp3^+^ was equivalent between wild-type and Gpr83^−/−^ CD4^+^ T cells (Fig. 5b). Moreover, the frequency of CD4^+^ CD25^+^ cells that were Foxp3^+^ was also comparable (wild type, 86·4 ± 1·7%; Gpr83^−/−^, 81·1 ± 6·5%). Within the colon, no differences were observed in the frequencies of CD4^+^ T cells isolated from the lamina propria of wild-type and Gpr83^−/−^ mice (data not shown), and the frequencies of CD25^+^ and Foxp3^+^ cells within this population were similar. Again, the percentage of CD4^+^ CD25^+^ Foxp3^+^ T cells was also equivalent between the two groups (wild type, 90·2 ± 3·1%; Gpr83^−/−^, 93·4 ± 3·5%). These results indicate that Gpr83 does not play an integral role in the development of nT_R_ cells, nor in the peripheral homeostasis or tissue distribution of these cells under physiological conditions.

### Gpr83-deficient bone marrow cells contribute equivalently to the peripheral lymphocyte pool

As the effects of gene deficiency are often revealed in a competition assay with wild-type cells, we next assessed the ability of Gpr83^−/−^ bone marrow to compete with wild-type bone marrow in repopulating the peripheral T-cell compartment. BALB/c Ly9.2 RAG2^−/−^ recipients were sublethally irradiated (5.5 Gy) and transferred at a 1 : 1 ratio of wild-type Ly9.2^+^ bone marrow to Gpr83^−/−^ Ly9.1^+^ bone marrow cells. Ten weeks after transfer, mice were killed and the distribution of wild-type and Gpr83-derived T cells analysed. No differences were observed in the ability to reconstitute the peripheral lymphocyte compartment between bone marrow cells lacking Gpr83 expression and wild-type bone marrow, as assessed by the distribution of Ly9.1^+^ and Ly9.2^+^ CD4^+^, CD8^+^ and B220^+^ cells (data not shown).

**Figure 6:**
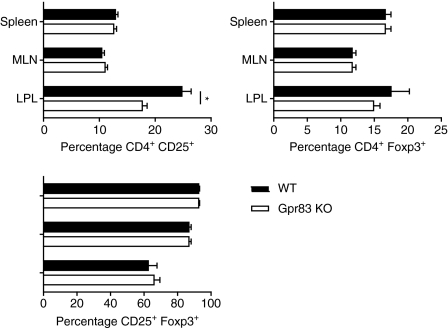
Mixed bone marrow chimera analysis reveals normal development and distribution of regulatory T (T_R_) cells from bone marrow cells deficient in the G-protein coupled receptor Gpr83. Irradiated (5·5 Gy) BALB/c Ly9·2 congenic RAG2^−/−^ recipients were injected with BALB/c Ly9·2^+^ and Gpr83-deficient Ly9·1^+^ bone marrow cells (5 × 10^6^ cells/mouse, 1 : 1 ratio). Mice were killed 10 weeks after transfer. Spleen, mesenteric lymph node (MLN) and colonic lamina propria (LPL) cells were isolated and analysed for expression of Ly9·1, CD25 and forkhead/winged helix transcription factor (Foxp3) in the CD4^+^ population. Data represent the mean of six individual mice ± standard deviation. KO, knockout.

The CD4^+^ T cells isolated from the mixed bone marrow chimeras were assessed for the expression of the nT_R_-cell markers CD25 and Foxp3. Within the spleen and MLNs, the percentages of wild-type (Ly9.2^+^) derived and Gpr83^−/−^ (Ly9.1^+^) derived CD4^+^ T cells expressing CD25 and Foxp3 (Fig. 6) were equivalent; however, there was a significant reduction (*P*= 0·0052) in the percentage of CD4^+^ CD25^+^ T cells derived from Gpr83^−/−^ Ly9·1^+^ bone marrow in the lamina propria (Ly9.2^+^ CD4^+^ CD25^+^, 24·8 ± 3·7%; Ly9.1^+^ CD4^+^ CD25^+^, 17·7 ± 1·9%). The differences appeared to affect a Foxp3^−^ CD4^+^ CD25^+^ population as there was no significant difference in the percentage of CD4^+^ Foxp3^+^ cells or in the frequency of CD4^+^ CD25^+^ cells that were also Foxp3^+^ (Fig. 6).

### Gpr83 is not required for the T_R_-cell-mediated control of colitis

**Figure 7:**
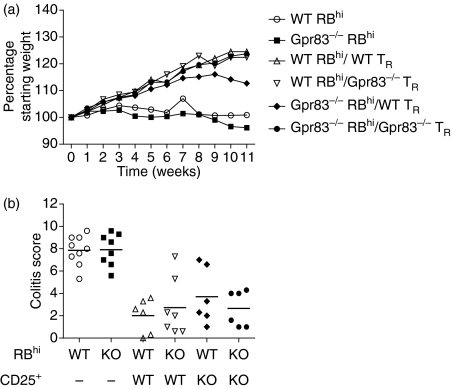
Regulatory T (T_R_) cells deficient in G-protein coupled receptor Gpr83 suppress colitis in a T-cell transfer model of colitis. BALB/c RAG2^−/−^ recipient mice were injected with 4 × 10^5^ CD4^+^ CD45RB^hi^ T cells isolated from either BALB/c or Gpr83-deficient mice either alone or in combination with 2 × 10^5^ CD4^+^ CD45RB^lo^ CD25^+^ wild-type (WT) or Gpr83-deficient T_R_ cells. (a) Mean weight loss/group; (b) colitis scores determined from histological analysis of colon sections. The data shown represent mean scores from individual mice pooled from two independent experiments. KO, knockout.

No obvious deficiencies could be detected in either the development of nT_R_ cells or their function in maintaining intestinal homeostasis under normal conditions. However, it was possible that in a more inflammatory setting there may be a requirement for Gpr83. In order to more fully assess the functional capacity of T_R_ cells lacking Gpr83 expression, we used a T-cell transfer model of colitis, in which a severe T helper type 1 (Th1)-mediated inflammation within the colon is induced following the transfer of naïve CD4^+^ CD45RB^hi^ T cells into immunodeficient recipients.^24^ The co-transfer of CD4^+^ CD45RB^lo^ CD25^+^ T_R_ cells is sufficient to prevent the development of disease. Wild-type BALB/c or Gpr83^−/−^ CD4^+^CD45RB^hi^ cells were transferred into RAG2^−/−^ BALB/c recipients either alone or in combination with wild-type or Gpr83^−/−^ CD4^+^ CD45RB^lo^ CD25^+^ T_R_ cells. Disease progression was monitored by weight loss and colitis assessed by histological analysis of colon tissue sections. Wild-type and Gpr83^−/−^ CD4^+^ CD45RB^hi^ T cells induced disease in recipient mice with no observable differences in onset or severity (Fig. 7). In addition, wild-type and Gpr83^−/−^ CD4^+^ CD45RB^lo^ CD25^+^ T_R_ cells were able to effectively suppress the development of disease induced by either wild-type or Gpr83^−/−^ naïve T cells as assessed by histological analysis of tissue isolated from the colon (Fig. 7). These results indicate that Gpr83 expression in nT_R_ cells is dispensable for their ability to regulate colitogenic T cells *in vivo*.

## Discussion

Gene expression profiling has identified a number of genes whose expression is highly enriched within the CD4^+^ Foxp3^+^ T_R_-cell subset. Amongst these, the orphan G-protein coupled receptor Gpr83 has been associated with the nT_R_-cell phenotype. Here we have analysed the expression of Gpr83 and produced Gpr83-deficient mice to critically examine its role in T_R_-cell development and function. Our results show that Gpr83 expression is tightly linked to Foxp3 expression and that, like Foxp3, its expression in antigen-activated cells is highly TGF-β dependent. Despite this association with Foxp3, Gpr83 appears to be dispensable for nT_R_-cell development and function. Thus Gpr83-deficient mice had normal frequencies of CD4^+^ Foxp3^+^ cells in the primary and secondary lymphoid organs and these cells retained their suppressive activity *in vivo* in a T-cell transfer model of colitis.

Previous studies suggest Gpr83 expression is restricted to Foxp3^+^ T cells and is not induced upon activation.^23^ By contrast to these results, we found a striking up-regulation of Gpr83, equivalent to levels found amongst nT_R_ cells, following polyclonal activation of naïve CD4^+^ T cells. Importantly, this induced expression was not associated with a general activation phenotype but was strongly linked with Foxp3, as Gpr83 expression was only detected in activated cells also expressing Foxp3. Furthermore, Gpr83 expression is highly dependent on TGF-β, a cytokine known to induce functional regulatory (iT_R_) cells from naïve CD4^+^ T cells *in vitro*.^5^ Whether TGF-β is acting directly to induce Gpr83 or whether this is a secondary event following induction of Foxp3 is unclear. A recent paper by Hansen *et al.* has described the preferential induction of Foxp3 in cells retrovirally transduced with Gpr83.^23^ This induction occurred under inflammatory conditions, and suggests that Gpr83 may be involved in ‘conditioning’ cells to express Foxp3 and adopt a regulatory phenotype. However, there does not appear to be an absolute requirement for Gpr83 in the peripheral induction of Foxp3 *in vitro*, as addition of TGF-β to activated Gpr83-deficient T cells led to similar Foxp3 expression as was observed with wild-type T cells (C. Toms and F. Powrie, unpublished observations).^25^ Further experiments are required to clarify a role for Gpr83 in peripheral induction of Foxp3 expression.

TGF-β signalling also sustains Gpr83 expression in nT_R_ cells, as there was a decrease in Gpr83 expression in nT_R_ cells isolated from dnTGF-βRII mice. By contrast, the expression of Foxp3 was comparable between wild-type and dnTGF-βRII nT_R_ cells, suggesting that the latter population does not contain significantly increased numbers of activated non-T_R_ cells, which would reduce the overall expression of Foxp3 within this population. This observation also implies a differential sensitivity of Foxp3 and Gpr83 to TGF-β signalling in nT_R_ cells, especially in the periphery of these mice. It is also notable that Gpr83 expression is highly dependent on IL-2, as nT_R_ cells isolated from mice lacking IL-2 expression show significantly reduced levels of Gpr83.^16^ Further studies on the function of Gpr83 would be aided by the identification of the specific ligand for this protein. Preliminary studies have suggested that this may be provided by antigen-presenting cells,^25^ although further work is required to definitively demonstrate this. Sequence analysis has revealed that Gpr83 shares the highest sequence homology with members of the neuropeptide Y receptor family,^26^,^27^ and a recent study demonstrated functional binding of Gpr83 to neuropeptide Y peptide analogues.^28^ The *in vivo* relevance of this has yet to be determined, although there is now increasing evidence for interaction between the neurological and immunological systems within the body.

Despite its strong link to Foxp3 expression, there were no developmental or functional defects in nT_R_ cells derived from mice deficient in Gpr83 expression. These cells could be detected at normal frequency within the thymus and periphery, and the lack of inflammatory disease in these mice suggests no functional abnormalities in nT_R_ cells under steady-state conditions. Similar results have been obtained by Lu *et al.*^25^ However, unlike that study we did find a modest, but significant splenomegaly in mice lacking Gpr83, although the cause or relevance of this remains unclear.

Foxp3^+^ T_R_ cells play a key role in maintaining intestinal homeostasis and this probably involves the activity of thymic-derived and peripherally induced T_R_-cell populations. Using a T-cell transfer model of colitis we found that CD4^+^ CD25^+^ T_R_ cells from Gpr83-deficient mice were able to suppress the development of colitis induced by wild-type or Gpr83^−/−^ CD45RB^hi^ naïve CD4^+^ T cells. This result demonstrates, in this *in vivo* disease model at least, that nT_R_ cells can regulate inflammation in the absence of Gpr83 expression. As Gpr83 is up-regulated in TGF-β-induced Foxp3 iT_R_ cells, it was possible that functional differences in the pathogenicity of Gpr83-deficient CD4^+^ CD45RB^hi^ T cells would be observed. However, no detectable differences were seen in either the onset or severity of disease induced by wild-type or Gpr83-deficient colitogenic T cells. Comparable levels of Foxp3 could be detected in the CD4^+^ T cells isolated from CD45RB^hi^ transferred mice, suggesting that there was no defect in the ability to induce Foxp3 in these cells upon transfer (data not shown). Taken together, our data indicate that Gpr83 expression is dispensable for nT_R_ cell function and control of intestinal homeostasis. However, the finding that Gpr83 is a TGF-β-responsive gene highlights a potential role in the peripheral development of Foxp3^+^ T_R_ cells. Further studies are required to establish this link.
